# Causal association between mTOR-dependent circulating protein levels and central precocious puberty: a Mendelian randomization study

**DOI:** 10.3389/fendo.2024.1360043

**Published:** 2024-03-07

**Authors:** Yuanxiao Ying, Ze Yu, Liping Wu

**Affiliations:** ^1^ Department of Pediatrics, Zhoushan Hospital, Wenzhou Medical University, Zhoushan, China; ^2^ Laboratory of Cytobiology & Molecular Biology, Zhoushan Hospital, Wenzhou Medical University, Zhoushan, China; ^3^ Science and Education Section, Zhoushan Hospital, Wenzhou Medical University, Zhoushan, China

**Keywords:** causal relationship, central precocious puberty, eIF4G, Mendelian randomization, mTOR

## Abstract

**Background:**

The mechanistic target of rapamycin (mTOR) signaling pathway has a significant effect on central precocious puberty (CPP). However, the causality between mTOR-dependent circulating protein levels and CPP is still unclear. Our aim is to assess the effects of seven mTOR-dependent circulating protein levels on CPP using Mendelian randomization (MR).

**Methods:**

Instrumental variables (IVs) for mTOR-dependent circulating protein levels were retrieved from the proteomics-GWAS INTERVAL study and eQTLGen. The summary-level genetic datasets for CPP outcome were obtained from the FinnGen Consortium. Inverse-variance weighted (IVW) was used as the primary method and the pleiotropy, heterogeneity and robustness of the analyses were detected as sensitivity analysis. Positive exposures in the discovery cohort would be revalidated in the validation cohort.

**Results:**

This two-sample MR study revealed a causal association between eIF4G level in plasma and CPP in both discovery cohort (IVW: OR = 0.45, 95% CI = 0.22–0.91, *p* = 0.026) and validation cohort (IVW: OR = 0.45, 95% CI = 0.24–0.85, *p* = 0.014).

**Conclusions:**

There was a causal association between eIF4G level in plasma and CPP. Whether eIF4G can be used for the prevention or treatment of CPP needs to be explored in further studies.

## 1 Introduction

Central precocious puberty (CPP) is a pediatric endocrine disorder caused by premature activation of the hypothalamic-pituitary-gonadal axis that leads to early puberty in children (before 8 years of age for girls and 9 years of age for boys) (1), and is the main type of precocious puberty (2). In Denmark, a statistic based on a registered population showed that the prevalence of precocious puberty was about 0.2% in girls and less than 0.05% in boys (3). Notably, precocious puberty can affect adult height and increase the risk of behavioral and psychological problems, cancer, obesity, cardiovascular disease and type 2 diabetes (4–8). Therefore, the prevention of precocious puberty is particularly important in global public health.

The mechanistic target of rapamycin (mTOR) is a serine/threonine protein kinase in the PI3K-related protein kinase (PIKK) family and participates in the composition of two protein complexes named mTOR complex 1 (mTORC1) and mTOR complex 2 (mTORC2) (9). mTORC1 performs essential functions in the regulation of cellular anabolism and catabolism, such as promoting the synthesis of proteins, lipids and nucleotides, maintaining energy homeostasis, and inhibiting catabolism and autophagy (9). In terms of protein synthesis, mTORC1 phosphorylates two key effectors downstream named eukaryotic translation initiation factor 4E binding protein (eIF4EBP) and ribosomal protein S6K kinase 1 (S6K1) to promote translation (10). Wherein phosphorylated eIF4EBP enhances 5′ cap-dependent mRNA translation by releasing eukaryotic translation initiation factor 4E (eIF4E) and promoting its formation of eukaryotic translation initiation factor 4F (eIF4F) with eukaryotic translation initiation factor 4A (eIF4A) and eukaryotic translation initiation factor 4G (eIF4G) (11). As for mTORC2, it regulates a variety of cellular structures and functions such as cytoskeleton, metabolism, proliferation and survival by phosphorylating members of the AGC (PKA/PKG/PKC) family of protein kinases including protein kinase C-alpha (PKC-α), serum- and glucocorticoid-induced protein kinase 1 (SGK1) and Akt (9, 12–14).

Previous studies have found that the mTOR signaling pathway has a significant effect on CPP. Activation of the IGF-1/Akt/mTOR pathway in the hypothalamus of prepubertal female rats increases the expression of kisspeptin protein (encoded by the Kiss-1 gene) and gonadotropin-releasing hormone (GnRH), resulting in an earlier puberty in female rats (15, 16). In contrast, inhibition of mTOR in the hypothalamus blocks the activation of Kiss-1, G-protein-coupled receptor GPR54 (also named Kiss1R) and GnRH (15). However, due to the lack of exploration of the relationship between downstream proteins in the mTOR signaling pathway and CPP in previous studies, the relationship between mTOR-dependent downstream proteins and CPP is currently inconclusive.

Mendelian randomization (MR) is an approach of genetic epidemiology that uses genetic variants, known as single nucleotide polymorphisms (SNPs), as proxies for exposures of interest to explore causal relationships between exposures and outcomes (17). The advantages of MR lie in the fact that it minimizes confounders, bias, and reverse causation associated with observational studies, and is consistent with the principle of randomization in randomized controlled trials (RCTs) (17). Given the important role of the mTOR signaling pathway in CPP, in the present study, we explored the effects of mTOR-dependent circulating protein (Akt, eIF4A, eIF4E, eIF4EBP, eIF4G, PKC-α and S6K1) levels on CPP using two-sample MR.

## 2 Materials and methods

### 2.1 Study design and MR assumptions

**Figure 1:**
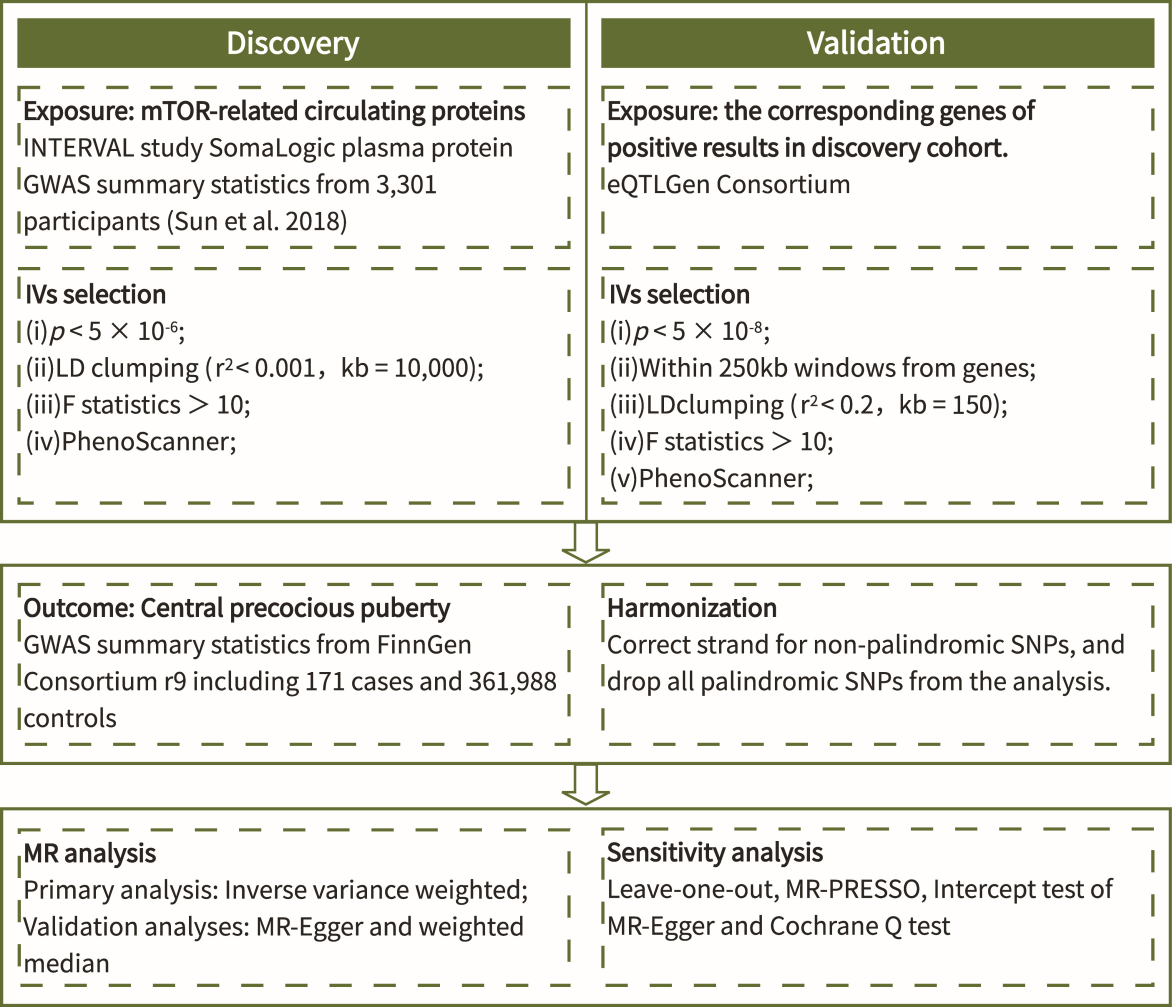
Flow chart of the study.

The process of this study was carried out in strict accordance with the Strengthening the Reporting of Observational Studies in Epidemiology Using Mendelian Randomization (STROBE-MR) Statement (18). The flow chart (**Figure 1**) shows the general design of this study.

**Figure 2:**
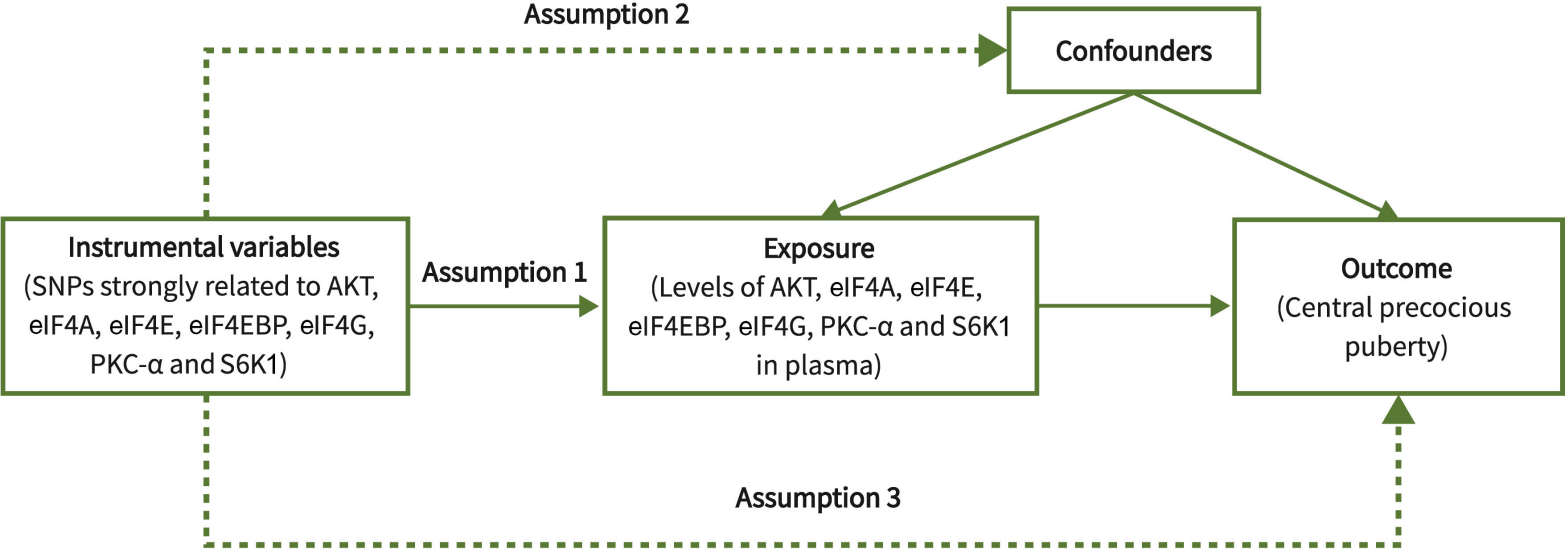
Three core assumptions of Mendelian randomization.

There are three assumptions that must be met in MR studies (**Figure 2**): (i) Genetic variants used to proxy exposure must be closely correlated with the exposure; (ii) Genetic variants are not associated with any confounders that affect the exposure-outcome relationship; (iii) Genetic variants can only impact outcomes via the target exposure. The rationale for the ability of MR studies to reduce bias is that genetic variants are randomly arranged and fixed at the time of conception, and are therefore largely unaffected by confounding factors and are not altered by disease progression.

### 2.2 Data sources for exposure and outcome

The genome-wide association study (GWAS) datasets of mTOR-dependent circulating protein levels in the discovery cohort were retrieved from a publicly available proteomics-GWAS INTEVAL study, which enrolled 3,301 participants of European ancestry and measured data on 3,600 proteins (19). The cis-eQTLs of corresponding genes of the mTOR-dependent circulating proteins in the validation cohort were retrieved from eQTLGen Consortium (20). The GWAS dataset of CPP outcome including 171 cases and 361,988 controls of European ancestry were retrieved from the FinnGen Consortium (R9) (21).

### 2.3 Selection of instrumental variables

The IVs for exposure in the discovery cohort were screened for the following conditions: (i) *p* < 5×10^-6^; (ii) linkage disequilibrium (LD) clumping (r^2^ < 0.001, threshold 10,000kb). The IVs for exposure in the validation cohort were screened for the following conditions: (i) *p* < 5×10^-8^; (ii) ± 250kb of the gene location; (iii) weak LD (r^2^ < 0.2, threshold 150 kb) to clump extracted cis-eQTLs in order to maximize the strength of the instrument for each gene. Meanwhile, all IVs in the discovery and validation cohorts need to meet F statistics (beta^2^/se^2^) (22, 23) > 10 in order to minimize weak instrumentation bias (24). Moreover, we assessed whether SNPs in screened IVs were associated with known risk factors of CPP by the PhenoScanner V2 database (http://www.phenoscanner.medschl.cam.ac.uk/). Dessert consumption, obesity, sun exposure, vitamin D levels and sleep duration are considered confounders of CPP (25), and any SNPs associated with these confounders would be excluded. Finally, when harmonizing the data of exposure and outcome, we removed all palindromic SNPs.

### 2.4 Statistical analyses

The Inverse-variance weighted (IVW) method was considered as the primary method for determining the causal relationship between exposure and outcome in this study, which is found to be more reliable in the absence of pleiotropy of IVs (26). Meanwhile, MR-Egger (27) and weighted median (WM) (28) were used as complementary methods to verify the robustness of the results. MR-Egger regression provides MR estimates after adjustment for horizontal pleiotropy (27). When more than half of the information comes from valid instrumental variables, the WM approach produces estimates that are consistent with the actual effects (28). In this study, the results of IVW method were used as a criterion for determining whether there was a causal relationship or not, based on the premise that the results of all three methods had a consistent direction.

In addition, several sensitivity analyses were conducted to assess the pleiotropy, heterogeneity and robustness of the analyses. First, the Mendelian Randomization Pleiotropy RESidual Sum and Outlier (MR-PRESSO) (29) and the intercept test of MR-Egger (27) were applied to detect the horizontal pleiotropy, with *p* < 0.05 indicating the presence of horizontal pleiotropy. Second, the Cochrane Q test was applied to detect heterogeneity between genetic variants for the IVW and MR-Egger methods, where *p* < 0.05 indicated the presence of heterogeneity (30). Third, we visually assessed the heterogeneity among SNPs through the funnel plot. Fourth, we also performed leave-one-out sensitivity analyses to assess whether the results remained stable after excluding a particular SNP. Finally, we replicate the positive results from the discovery cohort in the validation cohort to demonstrate the robustness of the results.

All statistics were two-tailed, and *p* value < 0.05 was taken as strong evidence for the existence of a significant causal relationship. All statistical analyses were conducted using R version 4.3.1.

## 3 Results

### 3.1 Genetic instruments selection

After rigorous screening, the numbers of eligible SNPs as IVs for mTOR-dependent circulating protein levels were as follows in the discovery cohort: 14 for Akt, 8 for eIF4A, 13 for eIF4E, 10 for eIF4EBP, 7 for eIF4G, 8 for PKC-α and 11 for S6K1. In the validation cohort, 1, 3 and 13 eligible SNPs were selected as IVs for eIF4G1, eIF4G2 and eIF4G3. Detailed information of all IVs is displayed in **Supplementary Tables 1A, B**. **Supplementary Table 2** shows the strongly correlated phenotypic features corresponding to all SNPs used as IVs, none of which were excluded due to correlation with confounders of CPP.

### 3.2 Effect of mTOR-dependent circulating protein levels on CPP

**Figure 3:**
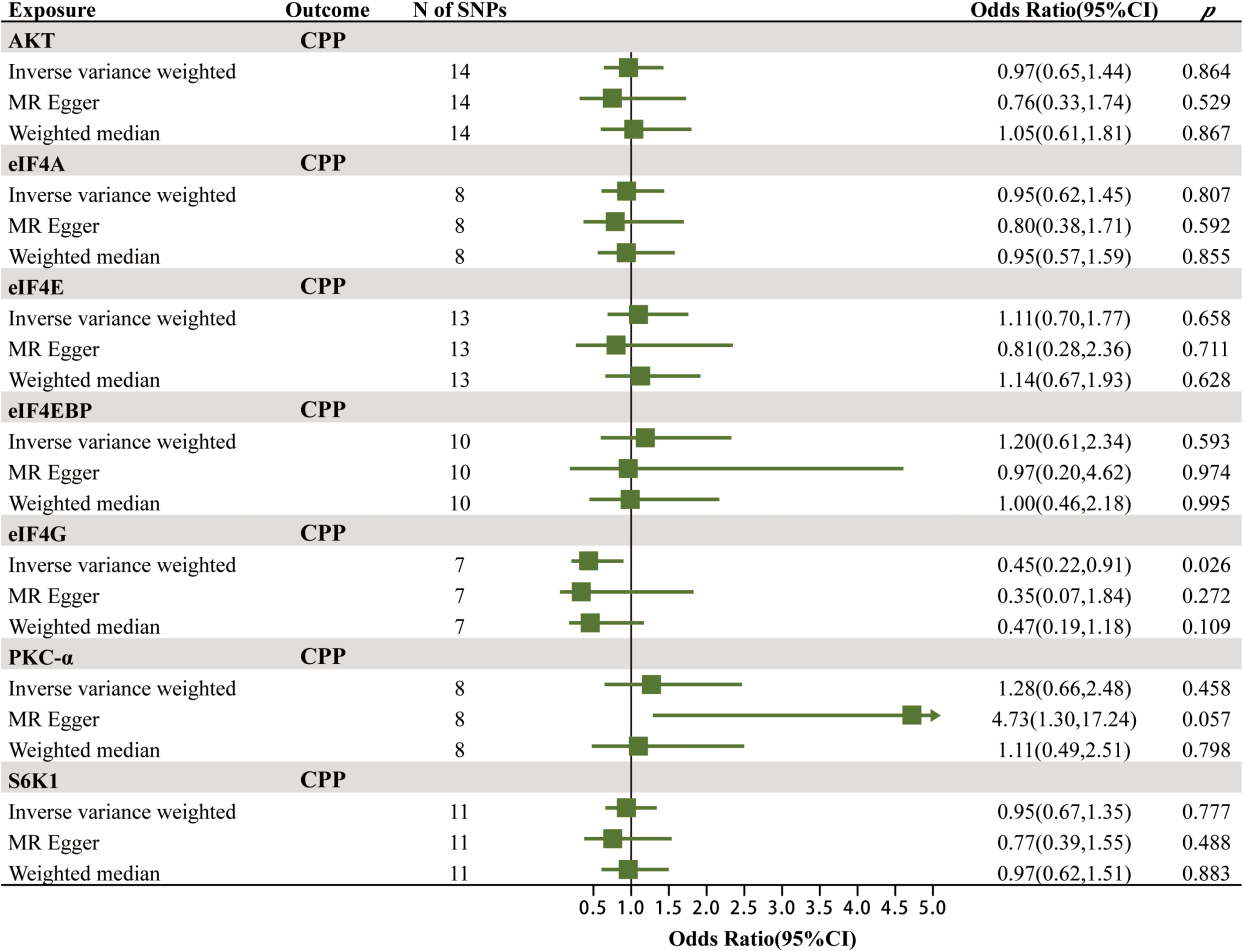
The MR results of the effects of seven mTOR-dependent circulating protein levels on CPP.

The MR results of the effects of seven mTOR-dependent circulating protein levels on CPP are summarized in **Figure 3**; **Supplementary Table 3**. Specifically, we found a significant causal relationship between the level of eIF4G in plasma and CPP. For every increase of the level of eIF4G in plasma by one standard deviation, the risk of CPP was reduced by 55% (IVW: OR = 0.45, 95% CI = 0.22–0.91, *p* = 0.026). However, no significant causality was found between the remaining six mTOR-dependent circulating protein levels and CPP.

**Figure 4:**
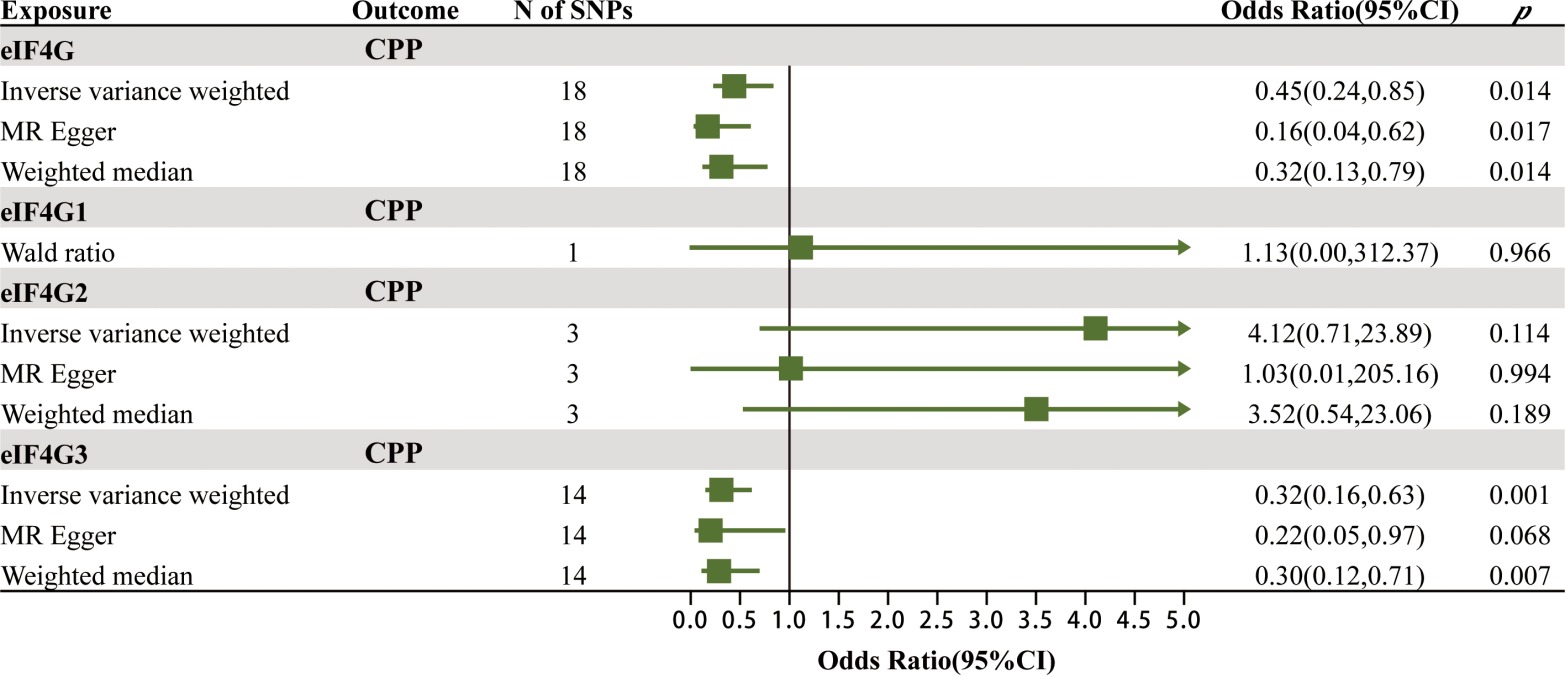
The MR results of the effects of gene eIF4G levels in the blood on CPP.

To verify the robustness of the above positive findings, we further explored the association between the levels of gene eIF4G in the blood and CPP. Protein eIF4G has three isoforms, named eIF4G1, eIF4G2 and eIF4G3, which are encoded by the genes eIF4G1, eIF4G2 and eIF4G3. The MR results of the effects of gene eIF4G levels in the blood on CPP are summarized in **Figure 4**; **Supplementary Table 4**. It was found that there was a significant causal relationship between the total level of gene eIF4G in the blood as well as the level of gene eIF4G3 and CPP. For every increase of the total level of gene eIF4G and the level of gene eIF4G3 in the blood by one standard deviation, the risk of CPP was reduced by 55% (IVW: OR = 0.45, 95% CI = 0.24–0.85, *p* = 0.014) and 68% (IVW: OR = 0.32, 95% CI = 0.16–0.63, *p* = 0.001), respectively. While no significant causality was found between the level of gene eIF4G1 in the blood as well as eIF4G2 and CPP. Such results were consistent with the discovery cohort, and furthermore, it was identified that it was gene eIF4G3 that played a major role in preventing CPP.

### 3.3 Sensitivity analysis

The results of MR-PRESSO, intercept test of MR-Egger and the Cochrane Q test in the discovery and validation cohorts are presented in **Supplementary Table 3**, **Supplementary Table 4**, respectively. It was confirmed by MR-PRESSO and intercept test of MR-Egger results (*p* > 0.05) that there was no horizontal pleiotropy in all analyses of this study. Meanwhile, all the Cochrane Q test results were negative (*p* > 0.05) and no significant asymmetry was found in the funnel plot (**Supplementary Figure 1**, **Supplementary Figure 2**), which suggested that there was no significant heterogeneity among the SNPs used as IVs in this study. Finally, as demonstrated by the results of the leave-one-out analysis (**Supplementary Figure 3**, **Supplementary Figure 4**), none of the SNPs significantly affected the causality between the positive exposures (the level of circulating protein eIF4G, the total level of gene eIF4G in the blood and the level of gene eIF4G3 in the blood) and CPP, which further validated the robustness of the results.

## 4 Discussion

To our knowledge, this is the first study to assess the causal relationship between mTOR-dependent circulating protein levels and CPP using MR method. This MR study provided strong evidence of a significant negative correlation between mTOR-dependent circulating protein eIF4G level and the risk of CPP. However, there was no evidence of a causal relationship between the levels of the remaining mTOR-dependent circulating proteins (Akt, eIF4A, eIF4E, eIF4EBP, PKC-α and S6K1) and CPP.

Premature activation of the hypothalamic-pituitary-gonadal axis is the key to CPP, behind which the leptin-mTOR-Kisspeptin pathway serves as one of the crucial pathogenic mechanisms (31). In addition to suppressing appetite and increasing energy expenditure, leptin has also been implicated as a permissive factor in the activation of GnRH neurosecretion during puberty (32). Meanwhile, Kisspeptin is a key activator of gonadotropin secretion because it induces GnRH secretion by activating GnRH neurons via direct synaptic contact or indirect trans-synaptic inputs (33, 34). Roa et al. found that inhibition of central mTOR by rapamycin attenuates the permissive effect of leptin on the onset of puberty in food-restricted female rats, while pharmacological inhibition of mTOR results in a significant decrease in hypothalamic Kiss-1 expression (35). Therefore, mTOR acts as an energy sensor in this pathway, however, the specific molecular basis of this pathway remains unclear (31).

Zhou et al. found that phthalate exposure is associated with an increased incidence of precocious puberty in girls in a case-control study, and Shao et al. found that Di-(2-ethylhexyl) phthalate (DEHP) induces precocious puberty in female rats by upregulating the IGF-1/PI3K/Akt/mTOR signaling pathway (15). Several studies have also pointed out (including a RCT study) that the PI3K/Akt signaling pathway or Akt1 is a key target for the prevention or treatment of precocious puberty (36–40). However, no significant causality between Akt levels in plasma and CPP was found in our study. Such differences may arise from the different tissues examined (hypothalamus, serum, plasma or testis) as well as the fact that our study did not obtain GWAS data on the levels of different Akt isoforms in plasma, instead we explored the total levels of Akt in plasma. Two previous studies observed reduced levels of PKC expression in the hypothalamus of precocious puberty rats and in the hypothalamic GT1-7 neurons of mice after treatment with gut microbiota-derived short-chain fatty acids (SCFAs) and the traditional Chinese medicine Fuyou formula, respectively (41, 42). In our study, we did not find a causal relationship between PKC-α level in plasma and CPP, which could be attributed to differences between tissues or species. Roa et al. found that hypothalamic phosphorylated S6K1 (pS6K1) and phosphorylated S6 (pS6) are downregulated in rats with central mTOR inhibition by rapamycin, whereas the expression levels of S6K1 and S6 are unchanged (35). This is consistent with our findings, suggesting that S6K1 levels in the hypothalamus and blood may not be associated with mTOR pathway-mediated pubertal development or precocious puberty. There have been no clear reports in the past on the relationship between eIF4A, eIF4E, eIF4EBP as well as eIF4G and CPP.

We found a causal relationship between eIF4G level in plasma and CPP, suggesting that eIF4G may be a target for the prevention or treatment of CPP. Such a relationship may be related to the following facts: First, as one of the components of the eIF4F complex, eIF4G binds to eIF4E, which is separated from eIF4EBP, and functions to promote protein synthesis when the mTOR signaling pathway is activated (11). Secondly, Mezey et al. found that leptin increases the eIF4E-eIF4G complex in the liver of male rats (43). Moreover, Lynch et al. found that leptin secretion is in turn regulated by the mTOR signaling (phosphorylation of eIF4G, S6K1, rpS6, and eIF4EBP1) *in vitro* and *in vivo* (44). The onset of puberty involves complex metabolic changes, with increased anabolism being one of the manifestations, and at the same time leptin is one of the critical factors in CPP. Taken together, these evidences suggest a potential complex relationship between eIF4G and the leptin-mTOR axis as well as CPP. We consider that firstly eIF4G level in plasma can be used as an objective basis for assessing potential disease risk in children with high risk factors of CPP (lack of exercise, vitamin D deficiency, poor sleep quality, exposure to endocrine disruptors and international adoption). Secondly, the causal relationship between eIF4G and CPP provides new insights into the potential mechanisms of leptin-mTOR signaling pathway in CPP. Thirdly, eIF4G is promising as a new target for the prevention or treatment of CPP. We speculate that increasing eIF4G level in plasma can help to prevent CPP when prepubertal children experience high risk factors of CPP and even help to treat CPP. However, further work is needed to reveal the underlying pathophysiologic mechanisms. It is crucial to clarify the standardized reference values for eIF4G in plasma that have preventive or therapeutic significance for CPP, to develop eIF4G modulators that meet therapeutic goals, and to clarify the timing, manner, and dosage of eIF4G modulators through further trials.

As an integrator of puberty signals, sex-specific kisspeptin is a potential mechanism for the sexual dimorphism in the onset of puberty and incidence of idiopathic CPP in children (45). The hypothalamus in females expresses higher levels of kisspeptin than in males, while observational research has found that the incidence of idiopathic CPP is at least 10-fold higher in females than in males (45). Therefore, it is essential to differentiate males from females when exploring the mechanisms of CPP. However, due to the lack of summarized data on CPP across genders in the current studies, our study was not able to explore whether the mTOR signaling pathway or mTOR-dependent circulating proteins are equally shared in both males and females by subgroup analysis.

There are several strengths in this study. First, we used genetic instruments to proxy mTOR-dependent circulating protein levels, which minimized bias and confounders. Second, we conducted the analysis in terms of both protein expression levels as well as gene expression levels, which improved the robustness of our conclusions.

This study has some limitations at the same time. First, only part of mTOR-dependent downstream proteins was included in this study. mTOR downstream pathways are extensive and the relationship between other related proteins and CPP needs to be further explored in subsequent studies. Second, the genetic variants in this study all came from blood samples. Despite our attempts to obtain GWAS data for the corresponding proteins in hypothalamic tissues via GTEx Consortium, we were ultimately unable to obtain suitable instrumental variables. Third, the eQTLs and GWAS data are derived from European population, which makes our conclusions lack universality. Fourth, as mentioned above, CPP differs between males and females to some extent, and our study did not involve subgroup analysis of gender or different clinical picture. Fifth, the INTEVAL study included only 3,301 participants making it impossible to detect as many genome-wide important genetic variants as possible. Therefore, we chose the looser threshold *p* < 5×10^-6^ in the context of balancing statistical efficacy rather than the commonly used GWAS significance level *p* < 5×10^-8^. Sixth, the CPP outcome data from FinnGen Consortium has a relatively small number of cases and larger data of CPP are needed for further validation in the future.

## 5 Conclusion

This two-sample MR study supports a causal association between eIF4G level in plasma and CPP. eIF4G level in plasma can be used as an objective basis for assessing potential disease risk in children with high risk factors of CPP. However, whether eIF4G is involved in the leptin-mTOR signaling pathway in CPP and whether it can be used for the prevention or treatment of CPP need to be explored in further studies.

## Data availability statement

The original contributions presented in the study are included in the article/**Supplementary Material**. Further inquiries can be directed to the corresponding authors.

## Ethics statement

No additional ethical approval was required as all data for this study were retrieved from publicly available databases. The organizations that provide public data all obtained informed consent from the participants and approval from the relevant ethics committees before their study.

## Author contributions

YY: Formal analysis, Software, Writing – original draft. ZY: Formal analysis, Funding acquisition, Methodology, Writing – review & editing. LW: Conceptualization, Data curation, Project administration, Resources, Writing – review & editing.
