# Supplementary material for: Causal association between mTOR-dependent circulating protein levels and central precocious puberty: a Mendelian randomization study
Source: Front Endocrinol (Lausanne). 2024 Mar 7;15:1360043. doi: 10.3389/fendo.2024.1360043 (PMC10954777; doi:10.3389/fendo.2024.1360043)
Supplement: Supplementary file 2 [file Image_1.pdf]

Supplementary Material

Causal association between mTOR-dependent circulating protein levels and central precocious puberty: a Mendelian randomization study

Supplementary Figures

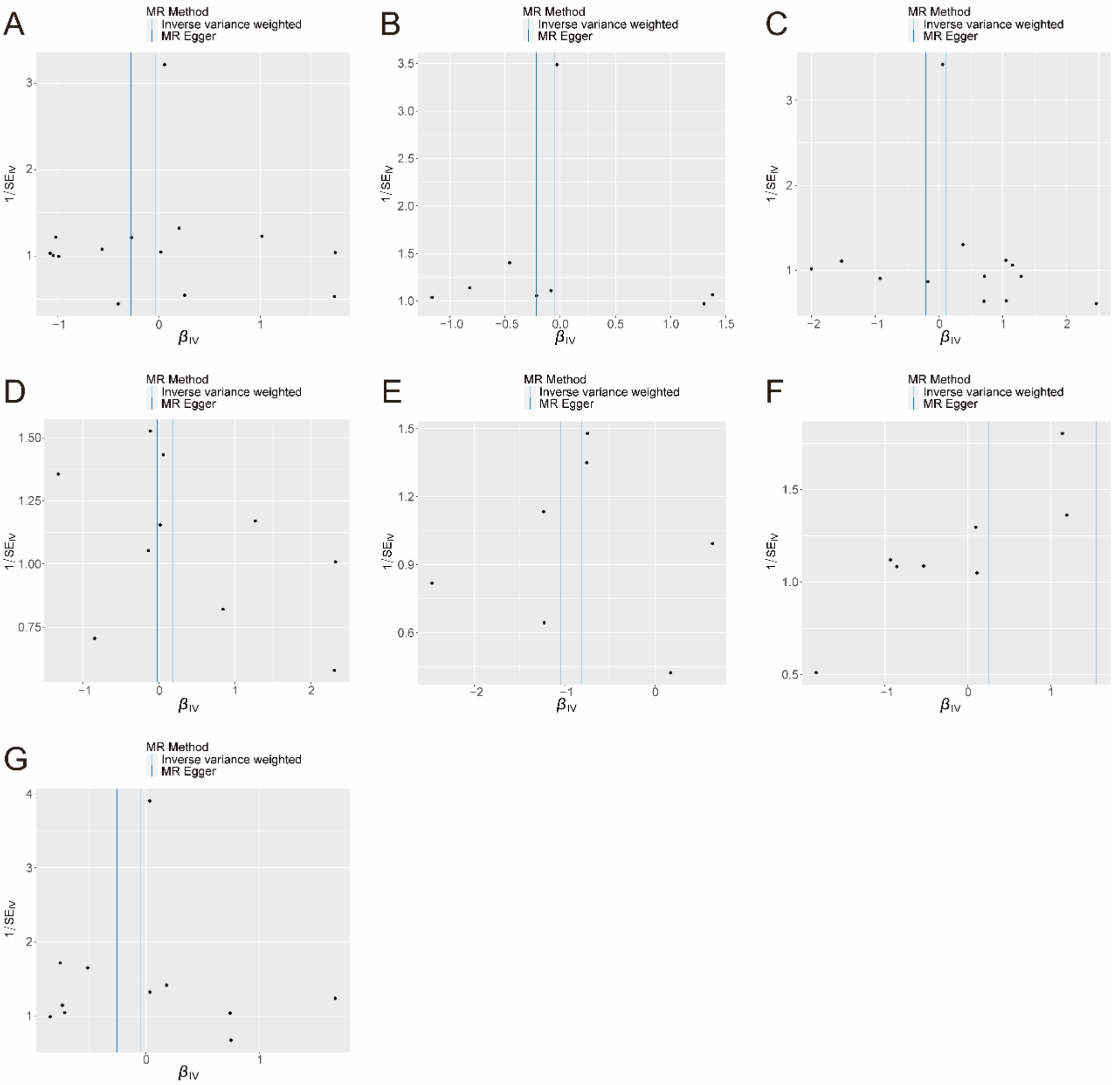

Supplementary Figure 1. Funnel plots for mTOR-dependent circulating protein levels on CPP to access bias. (A) Akt; (B) eIF4A; (C) eIF4E; (D) eIF4EBP; (E) eIF4G; (F) PKC-α; (G) S6K1.

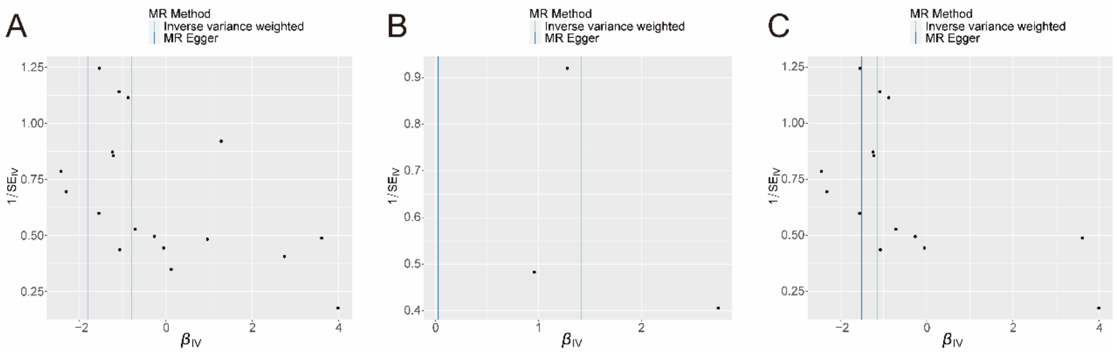

Supplementary Figure 2. Funnel plots for gene eIF4G levels on CPP to access bias. (A) eIF4G; (B) eIF4G2; (C) eIF4G3.

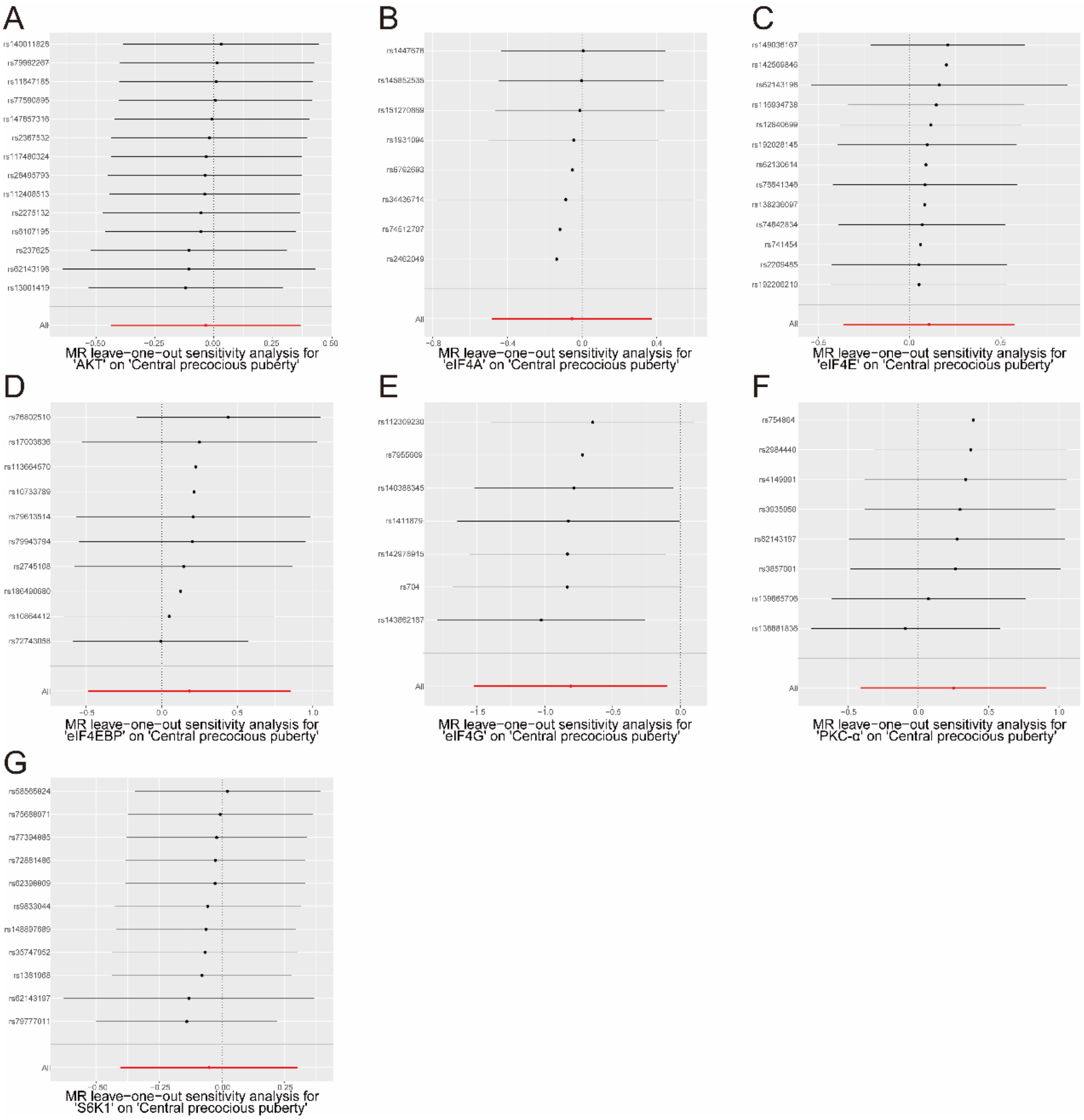

Supplementary Figure 3. Leave-one-out analysis for the estimates of mTOR-dependent circulating protein levels on CPP. (A) Akt; (B) eIF4A; (C) eIF4E; (D) eIF4EBP; (E) eIF4G; (F) PKC-α; (G) S6K1.

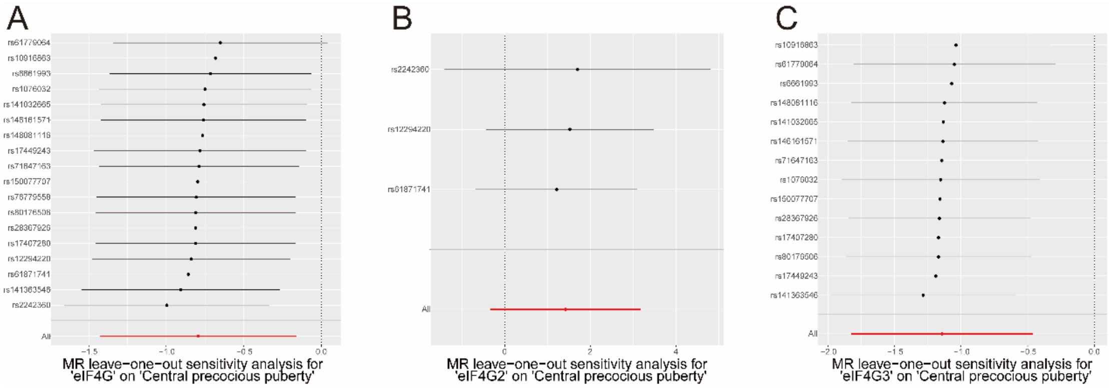

Supplementary Figure 4. Leave-one-out analysis for the estimates of gene eIF4G levels on CPP. (A) eIF4G; (B) eIF4G2; (C) eIF4G3.
